# Preharvest Application of Phenylalanine Induces Red Color in Mango and Apple Fruit’s Skin

**DOI:** 10.3390/antiox11030491

**Published:** 2022-02-28

**Authors:** Michal Fanyuk, Manish Kumar Patel, Rinat Ovadia, Dalia Maurer, Oleg Feygenberg, Michal Oren-Shamir, Noam Alkan

**Affiliations:** 1 Department of Postharvest Science of Fresh Produce, Agricultural Research Organization (ARO), Volcani Institute, Rishon LeZion 7505101, Israel; michal.fanyuk@mail.huji.ac.il (M.F.); patelm1402@gmail.com (M.K.P.); daliam@volcani.agri.gov.il (D.M.); fgboleg@volcani.agri.gov.il (O.F.); 2 Robert H. Smith Faculty of Agriculture, Food and Environment, The Hebrew University of Jerusalem, Rehovot 76100, Israel; 3 Department of Plant Science, Agricultural Research Organization (ARO), Volcani Institute, Rishon LeZion 7505101, Israel; rinat@volcani.agri.gov.il (R.O.); vhshamir@volcani.agri.gov.il (M.O.-S.)

**Keywords:** anthocyanin, phenylalanine, prohydrojasmon, flavonoids, mango, apples, preharvest, red color, fruit quality

## Abstract

Anthocyanins are secondary metabolites responsible for the red coloration of mango and apple. The red color of the peel is essential for the fruit’s marketability. Anthocyanins and flavonols are synthesized via the flavonoid pathway initiated from phenylalanine (Phe). Anthocyanins and flavonols have antioxidant, antifungal, and health-promoting properties. To determine if the external treatment of apple and mango trees with Phe can induce the red color of the fruit peel, the orchards were sprayed 1 to 4 weeks before the harvest of mango (cv. Kent, Shelly, and Tommy Atkins) and apple fruit (cv. Cripps pink, Gala and Starking Delicious). Preharvest Phe treatment increased the red coloring intensity and red surface area of both mango and apple fruit that was exposed to sunlight at the orchard. The best application of Phe was 2–4 weeks preharvest at a concentration of 0.12%, while a higher concentration did not have an additive effect. A combination of Phe and the positive control of prohydrojasmon (PDJ) or several applications of Phe did not have a significant added value on the increase in red color. Phe treatment increased total flavonoid, anthocyanin contents, and antioxidant activity in treated fruit compared to control fruits. High Performance Liquid Chromatography analysis of the peel of Phe treated ‘Cripps pink’ apples showed an increase in total flavonols and anthocyanins with no effect on the compound composition. HPLC analysis of ‘Kent’ mango fruit peel showed that Phe treatment had almost no effect on total flavonols content while significantly increasing the level of anthocyanins was observed. Thus preharvest application of Phe combined with sunlight exposure offers an eco–friendly, alternative treatment to improve one of the most essential quality traits—fruit color.

## 1. Introduction

Fruits and vegetables are lost or get a lower price due to poor appearance, quality problems, and consumer preferences [1]. Fruit is downgraded if it does not meet a very high standard of quality requirements, causing loss of profitability [2] and possibly food loss. Color and appearance, nutritional value, texture, and aroma are the main factors determining fruit and vegetable quality [3].

Fruit quality and maturity stage can often be indicated by the color of its skin [4]. The consumer prefers red-colored fruit. Therefore, the red color in fruit peel is a major contributor to the acceptance of fruit, which allows it to be priced higher and sold more easily [5]. Red color of fruit peel is important in terms of appearance and constitutes an advantage in resistance to pathogens and cold. For example, mango fruit exposed to sunlight developed red color, had higher anthocyanin concentration in the fruit peel, and was more resistant to pathogenic fungi including *Colletotrichum gloeosporioides, Alternaria alternata*, and *Lasiodiplodia theobromae* in comparison with green fruit from the same tree [6,7]. Red-colored mango fruit (cv. Shelly) is more resistant to cold than green fruit and, therefore, could be stored for more extended periods [6,8].

Anthocyanins are the main compounds responsible for the red coloring of mango and apple fruit peel [9]. Anthocyanins and flavonoids are natural compounds synthesized via the phenylpropanoid pathway, which have antioxidant and antifungal qualities, as well as health-promoting properties for humans [7,10]. Anthocyanins and flavonoids synthesis is induced in response to biotic or abiotic stresses, such as light, temperature, drought, and pathogen attacks [11,12].

Phenylalanine (Phe), the precursor of the phenylpropanoid pathway, is an aromatic amino acid existing naturally in plants and derived from the shikimate pathway [13]. Flavonoids, including anthocyanins and lignin, are secondary metabolites of the phenylpropanoid pathway, which is one of the main plant defense mechanisms [14]. Application of Phe to petunia, *Arabidopsis,* and cut chrysanthemum flowers, increased their resistance to *B. cinerea* by activating the phenylpropanoid pathway and flavonoids with antifungal activity [15,16]. Similarly, applying phenylalanine pre- or postharvest reduces decay in mango, avocado, strawberry, and citrus fruit, which is caused by various pathogenic fungi [17].

Red color induction methods, including application of plant growth regulators, in fruit have been studied for many years. In particular, previous studies have shown that mango and apple fruit have stronger red pigmentation and higher anthocyanin levels when exposed to direct sunlight, when grown outside of the tree canopy, compared with fruit from the inner parts of the canopy [6,18]. Pre-harvest application of harpin proteins or inactivated yeast treatments also showed an enhanced red color and anthocyanin accumulation [19,20]. Moreover, it was found that exogenic application of Prohydrojasmon (PDJ), methyl jasmonate (MJ), or Abscisic acid (ABA), along with direct sunlight exposure, promotes the phenylpropanoid pathway in mango fruit and contributes to the accumulation of red skin on the fruit [4,21].

Mango (cv. Kent, Shelly, and Tommy Atkins) and apple (cv. Cripps pink, Gala, Starking Delicious, and Anna) are fruits known to have light-red fruit peel and were chosen for this study. Our research goal is to determine if preharvest Phe application to mango and apple orchards will induce the red color in the fruit peel. Preharvest application of Phe could provide a healthy, safe, and relatively cheap means to increase fruit red color, reduce postharvest decay, and improve the profitability of the cultivar.

## 2. Materials and Methods

### 2.1. Plant Material, Preharvest Spray, Harvest, and Storage

The orchards used for this study were twenty-year-old mango orchards (cv. Kent and Shelly and Tommy Atkins), in Kibbutz Ravid (32°51′03″ N 35°27′52″ E; elevation +165 m), 15-years old apple orchards (cv. Cripps pink, Starking Delicious, and Gala) in Merom Golan (33°07′59″ N 35°46′33″ E; elevation +977 m), and a 15-years old apple orchard cv. Anna was grown in Arugot (31°44′5″ N 34°46′15″ E). Average-size mango trees (cv. Shelly and Kent) in the season of August 2020 (three trees per repetition, three repetitions per treatment) and apple trees (cv. Gala and Cripps Pink) in August 2020 and November 2020 (three trees per repetitions, four repetitions per treatment) were untreated (control) or sprayed with 0.12% phenylalanine (Phe) or with 0.2% prohydrojasmon (PDJ) on different weeks (1, 2, or 3) before harvest. The experiments were repeated on mango (cv. Shelly and Tommy Atkins) and apple (Cripps pink, Anna and Starking Delicious) fruit from June to November 2021. With the exception of ‘Starking Delicious’ apple cultivar, the mango and apple fruits were harvested from the outside of the tree canopy, which was exposed to direct sunlight. After harvest, control, and treated mango and apple fruits were transported (up to 2 h) to the ARO Volcani Center, Israel, and immediately stored at 12 °C and 2 °C for 21–28 days, respectively, followed by 7 days of shelf-life storage at 22 °C. Gala apple fruit was stored at 2 °C for 2 months, followed by 13 days of shelf-life storage at 22 °C. The fruits were evaluated at harvest, after cold storage, and after shelf life.

### 2.2. Measurements of Fruit Skin Color

#### 2.2.1. Red Color Evaluation

We assessed the percentage of the red-colored surface area of each fruit from individual mango and apple fruits in each treatment at various time points (at harvest and after shelf life). Apple fruits were also evaluated after cold storage. Furthermore, the intensity of the red color was graded according to an index (0–5; 0―no red color, 1―faint red color, and 5―very intense red color). Data were collected and analyzed from 14 and 28 mango and apple fruits, respectively, per treatment.

#### 2.2.2. Chroma Measurement

The skin color (Hue) was measured at the reddest point of 14 and 26 mango and apple fruits, respectively, for each treatment using a CR-400/410 Chromometer (Konica Minolta, Osaka, Japan). The hue angle (h°) measures color according to the wheel of colors, where 120° angle represents green color; 40°–60° angle represents orange-yellow color and 0°–40° represents red color. The transition between red color to green color is represented by a* value, where value of +60° correlates to full red color and –60° correlates to full green color.

#### 2.2.3. Estimation of Chlorophyll, Anthocyanins, and Flavonoids in Fruit Skin

Fruit peel fluorescence was evaluated to measure chlorophyll (SFR_R), flavonoid (FLAV), and anthocyanin (ANTH_RG) signals using a Multiplex III fluorescence detector (Force A, Orsay, France), based on fluorescence signal ratios between excitations and emissions correlated to these compounds. Data were collected and evaluated from 14 and 26 fruits of each treatment in mango and apple fruit, respectively.

### 2.3. Decay Evaluation

Stem end rot (SER), side decay, and total rotten fruits of mango and total decay of apples were evaluated after shelf-life storage, and apples (cv. Gala) were also evaluated after cold storage. We evaluated decay incidence (percent of fruit) and severity (index 0–10; 0―no decay, 1―mild decay; 5―moderate decay; 10―severe decay) for each treatment per box.

### 2.4. Extract Preparation

Fruit peel samples from each treatment were harvested and stored at −80 °C. Samples were ground (IKA A11 basic, Germany) in liquid N_2_, and 0.5 g were transferred into methanol solution (70%) for extraction and kept on the shaker overnight (250–300 rpm). The extraction mixture was centrifuged at 4100 rpm for 20 min at 20 °C (NF 8000R, Nuve, Turkey), and the supernatant was collected. The extraction was repeated. The collected supernatant liquid was concentrated in a CentriVap Concentrator (Labconco, Kansas, MO, USA) at room temperature until the extraction solution reached a volume of 0.5 mL. The concentrated samples were centrifuged at 12,000 rpm for 10 min at 20 °C, diluted 1:4 with distilled water, and stored at 4 °C until further use.

#### 2.4.1. DPPH Assay

The free radical scavenging activity was measured by 2,2′- diphenyl-1-picrylhydrazyl (DPPH) assay as previously described [22,23] with slight modifications. The stock solution was prepared (0.24% in absolute methanol) and diluted with methanol until the absorbance was 0.98 ± 0.02 at 517 nm. For each treatment, the diluted extracts (1:4) of mango and apple peels were mixed with 1 mL of DPPH solution and incubated for 10 min at room temperature. The absorbance was measured at 517 nm, and scavenging activities were calculated according to the following equation:

$$scavenging\;activity\;\left(\%\right)=\left[\frac{OD_{517\;control}-OD_{517\;treatment}}{OD_{517\;control}}\right]\times 100$$

#### 2.4.2. Total Phenolic Content

Total phenolic content of mango and apple fruit peels was evaluated with 0.2 N Folin–Ciocalteu (FC) reagent, and gallic acid was used as a reference standard [24,25]. For each treatment, the diluted mango and apple peel extracts were mixed with 750 µL of FC reagent and incubated for 5 min. 500 µL of sodium carbonate (Na_2_CO_3_, 75 gL^−1^) were added to the reaction mixture followed by 15 min incubation. The absorbance was measured at 760 nm and total phenolic content was calculated as mg gallic acid equivalent/g of fresh weight (gFW).

#### 2.4.3. Total Flavonoid Content

To determine the total flavonoid content, the diluted mango and apple peel extracts from each treatment were mixed with 200 µL of sodium nitrite (NaNO_2_, 5%) and incubated for 5 min at room temperature. Then, 200 µL of aluminum chloride (AlCl_3_, 10%) and 1.5 mL of sodium hydroxide (NaOH, 1 M) were added to the reaction mixture and absorbance was measured at 510 nm. The total flavonoid content was calculated as mg quercetin equivalent/gFW [26].

### 2.5. Flavonoid and Anthocyanin Characterization (HPLC)

Analysis was conducted on peels of mango (cv. Kent) and apple (cv. Cripps Pink) fruit after cold storage, to identify and quantify the flavonoids and anthocyanins in the fruit peel. 0.3 g of peel samples were used for extraction (2 mL, methanol:water:acetic acid, 11:5:1, *v/v*) [27]. Anthocyanins and flavonols were quantified as described by [28].

The experiment was conducted according to [29]. High-performance liquid chromatography (HPLC) (Shimatzu, Kyoto, Japan) equipped with an LC-10AT pump, an SCL-10A controller, and an SPD-M10AVP photodiode array detector. Extracts were loaded onto an RP-18 column (Vydac 201TP54) and separated at 27 °C with the following solutions: (A) H2O, pH 2.3, and (B) H2O:MeCN:HOAc (107:50:40), pH 2.3. Solutions were applied as a linear gradient from a ratio of 4:1 (A:B) to 3:7 over 45 min and held at a ratio of 3:7 for an additional 10 min at a flow rate of 0.5 mL/min. Anthocyanidins and flavonoids were identified by comparing both the retention time and the absorption spectrum at 250–650 nm with those of standard purified anthocyanins and flavonols (ChemFaces, Wuhan), China and Extrasynthase (Genay, France). Identification and quantification of anthocyanins and flavonols were done using reference standards, and concentrations were expressed as peak area/gram of FW.

### 2.6. Statistical Analysis

Data are represented as mean value ± standard error (SE). Multifactorial analysis of variance (One-way ANOVA, Tukey–Kramer HSD test) and Wilcoxon non-parametric comparison were performed using JMP (JMP Pro 15 software, SAS Institute, Cary, NC, USA). Different letters represent a statistically significant difference (*p* ≤ 0.05) among different treatments at the same time point.

## 3. Results

**Figure 1:**
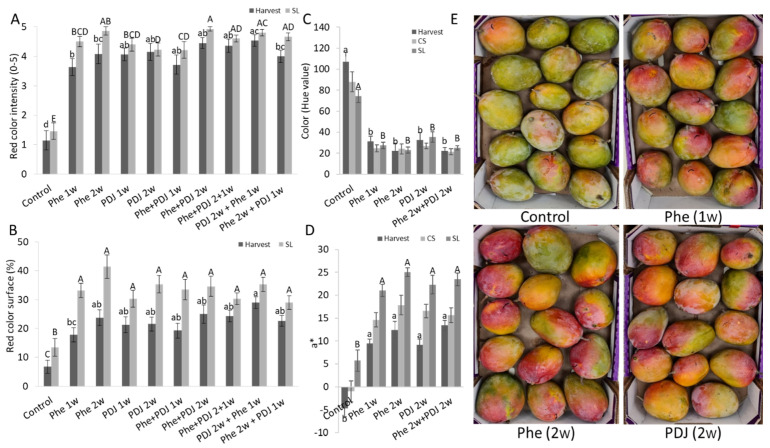
Red color evaluation of ‘Kent’ mango fruit peel. ‘Kent’ mango orchards were sprayed with 0.12% phenylalanine (Phe) or 0.2% prohydrojasmon (PDJ), one or two weeks preharvest. The fruit was evaluated at harvest (T0), after cold storage (CS, 3 weeks at 12 °C), and after shelf life (SL, 7 days at 22 °C). (**A**) Red color intensity (index 0–5). (**B**) Red surface area (% of fruit coverage). (**C**) Color (Hue value of the reddest point). (**D**) Green-red color range (a* value). (**E**) Representative pictures of ‘Kent’ mango boxes after shelf-life storage. Mean values and standard errors are presented. Statistical analysis was conducted for each time point separately (small or capital letters). Different letters represent a significant difference (*p* ≤ 0.05).

**Figure 2:**
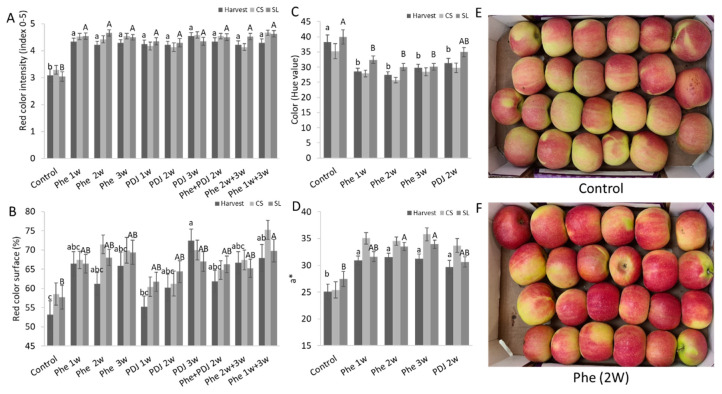
Red color evaluation of apple (cv. Cripps pink) peel. Apple orchard was sprayed preharvest with 0.12% phenylalanine (Phe) and/or 0.2% prohydrojasmon (PDJ). The fruit was evaluated at 3-time points: at harvest, after cold storage (CS, 3 weeks at 2 °C), and after shelf life (SL, 7 days at 20 °C). (**A**) Red color intensity (index 0–5). (**B**) Red surface area (% of fruit coverage). (**C**) Color (hue value at the reddest point). (**D**) Green-red color range (a* value at the reddest point). (**E**,**F**). Representative pictures of Cripps pink apple box after shelf-life storage. Mean values and standard errors are presented. Statistical analysis was conducted for each time point separately (small or capital letters). Different letters represent a significant difference (*p* ≤ 0.05).

The effect of preharvest Phe spraying on the redness of mango (cv. Kent and Shelly) and apple (cv. Cripps Pink and Gala) fruit peel was evaluated in comparison to untreated control fruit and the positive control of PDJ treated fruit. Fruit treated with either Phe or PDJ at harvest and after shelf-life storage had a significantly higher red color intensity of the peel in mango and apple fruit compared to the control (Figure 1A, Figure 2A, Figures S1A and S2A). The percentage of surface coverage of the red color in the mango (cv. Kent and Shelly) fruit peel at all evaluated time points was significantly higher in most treatments (Figure 1B and Figure S1B). Phe and PDJ significantly increased the red color surface coverage in almost all treated apples (cv. Gala) and increased the red color surface in treated ‘Cripps pink’ apples (Figure 2B and Figure S2B). Hue values at the reddest point on the fruit peel were evaluated at several time points (harvest, after CS, and after SL) for mango (cv. Kent and Shelly) and apple (cv. Cripps pink and Gala) fruit. In general, Phe and PDJ treatments decreased hue values (wheel of colors, where 120 is for green color; 0–40 represents red color) in almost all treated fruit (Figure 1C, Figure 2C, Figures S1C, S2C and S3A,C). This trend was especially apparent in mango (cv. Kent) and apple (cv. Gala) fruit, where hue values of treated fruit significantly decreased compared to control fruit both at harvest and after SL (Figure 1C, Figures S2A and S3A). In mango (cv. Shelly), fruit peel color varied similarly at harvest from 70.3 in control to 43.9–21.2 in treated fruit at this time point (Figure S1C). In apple (cv. Cripps pink), even though treated apples had significantly lower hue values at their reddest point compared to the control, all the treated and untreated fruit were within the orange color range (Figure 2C and Figure S3C). The Red-Green color range, which is represented by a* value, is directly proportional to the red-green color intensity. Higher and positive a* values correlate with more intense red color, whereas lower positive and negative values correlate with yellow and green color intensities, respectively. Phe and PDJ significantly increased a* values in most mango (cv. Kent and Shelly) and apple (cv. Gala) treated fruit. a* value of apple (cv. Cripps pink) also increased after Phe treatment compared to the control (Figure 1D, Figure 2D, Figures S1D, S2D, S3B,D).

The results described above were experiments conducted on apple cultivars (cv. ‘Anna,’ ‘Starking Delicious, and ‘Cripps Pink’) and mango cultivars (cv. ‘Shelly’ and ‘Tommy Atkins’) in the following year, which showed a similar trend (Tables S1–S3). The fruit treated preharvest with Phe showed a significant increase in red color intensity and red color surface of almost all treatments compared to control. Moreover, hue values generally decreased and a* value was usually increased (Tables S1 and S2).

Preharvest application of Phe in various concentrations showed that an increase in Phe concentration better induced the red color until optimum results at 0.12% Phe (Tables S1, S2 and S6). Phe at 0.12% increased the red color area and intensity in the fruit peel significantly better compared to lower concentrations as Phe at 0.01%, which was applied to apple (cv. Starking Delicious and Cripps pink) and mango (cv. Shelly) orchards (Table S1). Higher concentration (0.24%) did not contribute to better results of red color accumulation (Tables S1–S3). In the examination of the best time to apply Phe preharvest, it seems that in most experiments 2 weeks preharvest led to the best induction of red peel color (Tables S1–S3). While in Starking apples the application of 4 weeks preharvest was optimal for inducing red color (Table S4). A combination of several applications usually did not further induce the red color (Tables S2–S4). This induction of red color by preharvest application of Phe was correlated to a small decrease in decay incidence and severity in mango fruit (cv. Kent, Shelly, and Tommy) and inconclusive results in apple fruit (Tables S6 and S7).

**Figure 3:**
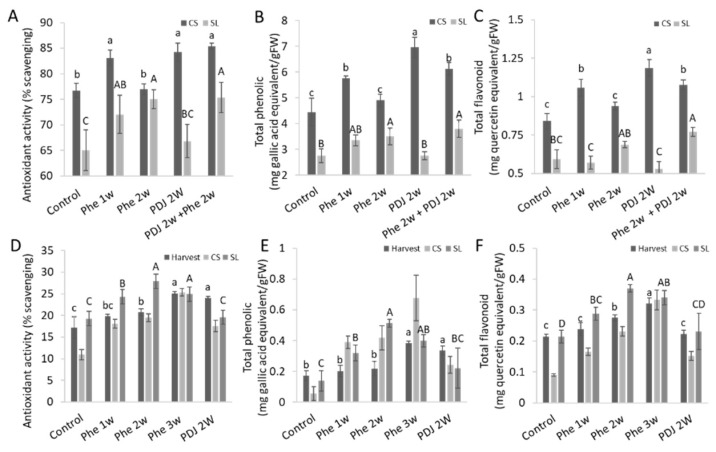
Effect of preharvest treatment with 0.12% Phe or 0.2% PDJ on antioxidant, phenolic, and flavonoid content in mango (cv. Kent) and apple fruit (cv. Cripps pink) at harvest, after cold storage (CS), and shelf-life (SL). Antioxidant activity, total phenolic, and total flavonoid content were evaluated from mango peels (cv. Kent) (**A**–**C**), apple (cv. Cripps pink) (**D**–**F**) peels. Mean values and standard errors are presented. Statistical analysis was conducted for each time point separately (small or capital letters). Different letters represent significant differences (*p* ≤ 0.05).

Preharvest application of Phe increased the antioxidant activity in mango (cv. Kent and Shelly) and apple (cv. Cripps pink) fruit peels (Figure 3A,D and Figure S4A). Phe also increased total phenolics and flavonoid content in most treatments in mango (cv. Kent and Shelly) and apple (cv. Cripps pink) fruit peels (Figure 3B,C,E,F and Figure S4B,C). Trends of total phenolic and flavonoid contents correlate to the level of antioxidant activity in mango fruit (cv. Kent and Shelly) both after CS and after SL, and in apple (cv. Cripps pink) after SL (Figure 3 and Figure S4). In both mango cultivars (cv. Kent and Shelly), combined treatment of PDJ 2w + Phe 2w had the highest antioxidant activity (Figure 3A and Figure S4A). However, in apples (cv. Cripps pink), Phe 2w and Phe 3w treatments had the highest antioxidant activity at harvest and after CS and SL, respectively (Figure 3D). The highest quantity of flavonoid content in mango (cv. Kent) was measured in the PDJ 2w treatment and PDJ 2w + Phe 2w treatment in mango (cv. Shelly) (Figure 3C and Figure S4C). Quantification of total phenolic content showed a similar trend in ‘Kent’ mango and ‘Shelly’ mango after CS (Figure 3B and Figure S4B). In apple (cv. Cripps pink), total phenolics and flavonoid content were highest in Phe 2w or 3w treatments, similar to its antioxidant evaluation (Figure 3).

**Figure 4:**
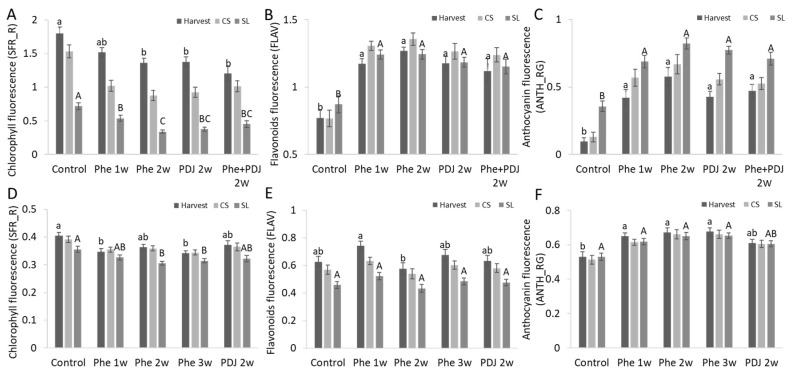
Effect of preharvest treatment of Phe and PDJ on chlorophyll, anthocyanin, and flavonoids fluorescence. Mango (cv. Kent, **A**–**C**) and apple (cv. Cripps pink, **D**–**F**) orchards were treated 1–3 weeks (1W, 2W, 3W) preharvest with 0.12% Phe or 0.2% PDJ and chlorophyll, anthocyanin, and flavonoids fluorescence at the reddest point of the fruit peel was analyzed at 3-time points: after harvest, after cold storage (CS), and after shelf-life (SL). (**A**,**D**) Chlorophyll fluorescence (SFR_R). (**B**,**E**) Flavonoids fluorescence (FLAV). (**C**,**F**) Anthocyanin fluorescence (ANTH_RG). Mean values and standard errors are presented. Statistical analysis was conducted for each time point separately (small and capital letters). Different letters represent significant differences (*p* ≤ 0.05).

The fluorescence of chlorophyll, flavonoids, and anthocyanins in the fruit peel was evaluated at the reddest point of all mango (cv. Kent and Shelly) and apple (cv. Cripps pink and Gala) fruit. Chlorophyll fluorescence decreased in almost all the Phe treated mango (cv. Kent and Shelly) and apple (cv. Cripps pink and Gala) fruits, and almost all treatments presented statistical significance (Figure 4A,D, Figures S5A,D and S6A,D).

‘Kent’ mango had a significant increase in flavonoid fluorescence in all Phe treatments compared to the control both after harvest and after SL (Figure 4B and Figure S6B). Similarly, ‘Shelly’ mango also showed an increase in flavonoids in all Phe treatments at harvest and after CS, however, after SL storage, no difference between the treatments was observed (Figure S6B). Apple (cv. Cripps pink and Gala) showed increased flavonoid fluorescence in treated fruit at harvest, while flavonoid fluorescence did not increase in treated fruit after SL storage (Figure 4F, Figures S5E and S6E). Phe and PDJ increased anthocyanin fluorescence in all treated fruit. The highest level of anthocyanins was detected mainly in Phe 2w and Phe 3w and their combination with other treatments in both mango (cv. Kent and Shelly) and apple (cv. Cripps pink) after SL. Apple (cv. Gala) presented the highest level of anthocyanins in Phe 1w treatment and its combinations (Figure 4C,F, Figures S5C,F and S6C,F).

**Figure 5:**
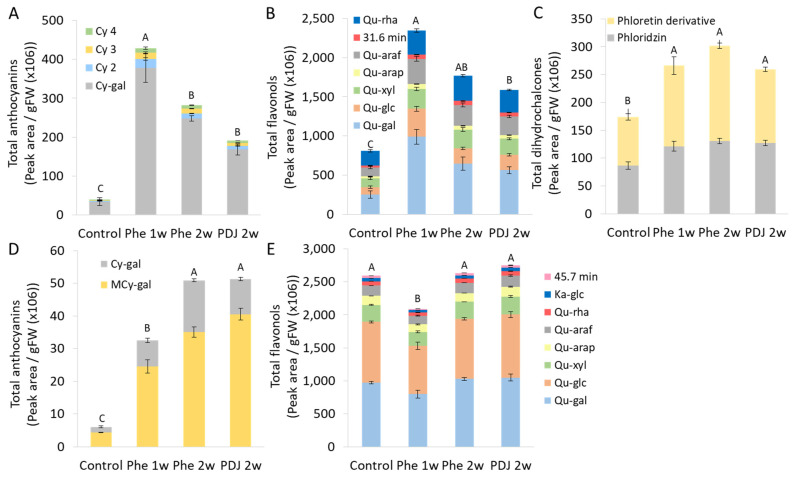
Quantification of metabolites using HPLC in mango and apple fruit peel. Mango (cv. Kent) and apple (cv. Cripps pink) were sprayed with 0.12% phenylalanine (Phe) or 0.2% prohydrojasmon (PDJ) one or two weeks preharvest. Apple (**A**–**C**) and mango (**D**,**E**) peels were analyzed after 3 weeks of cold storage at 2 °C and 12 °C, respectively. (**A**,**D**) Anthocyanin content in apple and mango, respectively. (**B**,**E**) Flavonol content in apple and mango, respectively. (**C**) Dihydrochalcones in apple. All the values expressed as Peak area/gFW (×10^6^). Mean values and standard errors are presented. Different letters represent significant differences for total content (*p* < 0.05).

Quantification and identification of metabolites in mango (cv. Kent) and apple (cv. Cripps pink) fruits treated with Phe or PDJ after cold storage was performed using HPLC analysis (Figure 5). In apple (cv. Cripps pink) peel, three types of compounds were detected related to the phenylpropanoid pathway: anthocyanins, flavonols, and dihydrochalcones, while in mango peel only anthocyanins and flavonols were detected (Figure 5). Both apple and mango peels from fruit treated with Phe had significantly higher levels of anthocyanins in the sample, compared to the control (Figure 5A,D). In mango, the anthocyanins content increased by 11.7 fold in the Phe 2w treatment. While the highest levels of anthocyanins and flavonols in apple (cv. Cripps pink) peel were observed in Phe 1w treatment, with an increase in anthocyanins and flavonols by 9.3 and 3.3 folds, respectively, compared to control (Figure 5). These levels of increase suggest a major shift in biosynthetic activity. Similarly, the total amount of dihydrochalcones detected in Phe treated apple samples was higher compared to the control (Figure 5C). Unlike mango, apple peel samples contained a significantly higher amount of flavonols in Phe treated fruit compared to the control (Figure 5B,E).

Eight flavonols were detected in mango (cv. Kent) peel: quercetin-3-*O*-galactoside (26.1 min, Qu-gal), quercetin-3-*O*-glucoside (27.2 min, Qu-glc), quercetin-3-*O*-xyloside (28.4 min, Qu-xyl), quercetin-3-*O*-arabinopyranoside (29.6 min, Qu-arap), quercetin-3-*O*-arabinofuranoside (30.4 min, Qu-araf), quercetin-3-*O*-rhamnoside (31.8 min, Qu-rha), kaempferol-3-*O*-glucoside (32.8 min, Ka-glc) and unknown compound (47.1 min) (Figure 5E). The ratios between the different flavonols within each treatment were similar, indicating that the treatment did not change the synthesis of individual compounds (Figure 5E and Figure S7). In apple peels, seven flavonols were revealed (Figure 5B and Figure S8). The chromatogram As opposed to mango (cv. Kent) peel analysis, several minor changes in composition levels were detected between treated fruit to control in apple (cv. Cripps pink) peel. For example, quercetin-3-*O*-galactoside (Qu-gal, 26.4 min) and quercetin-3-glucoside (Qu-glc, 27.6 min) showed a slightly higher level in treatments compared to the control, while quercetin-3-rhamnoside (Qu-rha, 32.3 min) and quercetin 3-xyloside (Qu-xyl, 28.8 min) showed lower levels in treated fruit compared to control. Most of the other compounds had a more or less similar ratio to the control (Figure 5B).

Mango contained only two anthocyanins: cyaniding-3-*O*-β-d-galactoside (11.3 min, Cy-gal) and 7-*O*-methylcyanidin 3-*O*-β-d-galactopyranoside (20.9 min, MCy-gal) (Figure 5D). 7-*O*-methylcyanidin 3-*O*-β-d-galactopyranoside was the main anthocyanin compound in mango fruit (Figure 5D and Figure S7). Anthocyanins apple (cv. Cripps pink) samples contained four cyanidin derivatives: cyanidin 3-galactoside (11.4 min, Cy-gal), and three unknown derivatives of cyanidin (18.0 min, 22.7 min, and 24.1 min) were detected. According to the literature, the most abundant peak in apple peels is cyanidin galactoside which has been identified before in ‘Cripps Pink’ [30,31]. The other cyanidin derivative peaks (Cy2, Cy3 and Cy4) in our study on apple peels could probably be designated as cyanidin 3-arabinoside, cyanidin 3-glucoside, cyanidin 3-xyloside [32,33]. All treatments presented similar ratios between the anthocyanin compounds, with cyanidin 3-galactoside being the main derivative detected (Figure 5). Dihydrochalcones, which were detected in apple peel as phloretin derivatives, also had a similar ratio in the control and treated fruit, including both phloridzin (35.1 min) and an unknown derivative of phloretin (31.7 min) (Figure 5C). The phloretin derivative could probably be designated as phloretin 2′-xyloglucoside [34]. In summary, it seems that preharvest Phe treatment increases the biosynthesis of flavonoids, anthocyanins, and dihydrochalcones, while keeping a similar ratio of the compounds as in the control (Figure 5).

## 4. Discussion

Red color is important for the marketability of fruit. Redder fruit can help decrease food loss and also contribute to customer health [10]. Red color of mango, apple, and other fruit peels is associated with the secondary metabolites named anthocyanins. In this work, we evaluated the effect of preharvest Phe application as a relatively cheap and eco-friendly method [17] on the induction of the red color of mango (cv. Kent, Tommy Atkins, and Shelly) and apple (cv. Starking Delicious, Gala and Cripps Pink) fruit peel exposed to direct sunlight. The main finding of this research is that preharvest application of phenylalanine in combination with sunlight radiation, results in the induction of anthocyanin biosynthesis, enhanced red color of the mango and apple fruit peel, and an increase in antioxidant activity.

Phenylalanine, a naturally occurring aromatic amino acid derived from the shikimate pathway [13], is the precursor for the phenylpropanoid biosynthetic pathway, where anthocyanins are among the secondary metabolites of the downstream pathway, which contribute to the accumulation of red color in the fruit peel [9]. Phe treatment increased flavonols and fragrance related to upstream of the phenylpropanoid pathway in various flowers but did not lead to the accumulation of anthocyanins and red color in flowers as well as Arabidopsis, tomato, chrysanthemum and petunia leaves [15,16,35]. Similarly, postharvest application to various fruits increased flavonols but did not increase the red color of fruit [17].

Flavonoid and phenylpropanoid pathways are defense mechanisms in the plant which are tightly regulated and induced in response to biotic or abiotic stress, that is, pathogens or sunlight [11,12]. Thus, Phe application induces flavonols production but does not increase the production of anthocyanins unless the downstream of the pathway is induced. Therefore, in this work, we show that preharvest treatment with phenylalanine on mango or apple fruit accompanied by sunlight radiation led to an induction of red color coverage and intensity of the peel. The increase in red coloration occurred in fruit on the outer side of the canopy, due to sunlight exposure. Indeed, sunlight radiation is known to induce the phenylpropanoid pathway in various plants, including mango and apple fruit [6,18,21,36].

The current study also applied prohydrojasmon (PDJ), an analog of Jasmonic acid phytohormone as a positive control, after having been described as effective in red color induction of the fruit peel [29]. Although Phe showed similar results as did PDJ, the mode of action of Phe seems to be as a precursor of phenylpropanoid pathway and not in a hormone-like manner. Phenylalanine also has an economic advantage that makes it more affordable compared to PDJ.

Previous studies reported that preharvest treatment of mango fruit (cv. Kent, Shelly, and Maya) with PDJ and ABA exposed to sunlight, increased the red color intensity and coverage of the fruit peel [21,29]. Similarly, both in apple and mango fruit, the red color surface coverage and intensity of the peel have increased due to the preharvest application of phenylalanine in combination with sunlight (Figure 1 and Figure 2). Shafiq and Singh have shown that phenylalanine at a concentration of 0.01% which was applied about 4 weeks preharvest increased the red color and anthocyanins of ‘Cripps Pink’ apples [37]. Here, various concentrations of Phe on both mango and apple fruit were tested on a larger scale in both mango and apples and at different years, finding that a higher concentration of 0.12% was much more efficient in inducing red color than the application of 0.01% of Phe (Tables S1, S3 and S4). It seems that increase concertation up to 0.12% led to an increase in red color in mango and apple peel (Tables S1 and S2).

The intensity of the red color, a*, and hue value measurements showed that Phe treated fruit at 0.12% had a redder and more intense red peel color, whereas the fruit in the control group had a lighter intensity red color varying from orange to yellow colors (Figure 1 and Figure 2). Similarly, preharvest applications of Methyl Jasmonate (MJ) and prohydrojasmon (PDJ) were reported to increase a* values and reduce hue values in mango fruit (cv. Mahachanok) [4,21].

The phenylpropanoid pathway is responsible for the synthesis of flavonoids and anthocyanins [38]. Preharvest treatment with Phe significantly increased anthocyanin levels both in mango (cv. Kent) and in apple (cv. Cripps pink) fruit, while flavonoid levels were either increased or unchanged (Figures S5 and S6). Further phenylpropanoid metabolic analysis was done by HPLC, which showed a significant increase in total anthocyanin and flavonols levels compared to control in apple (cv. Cripps pink) and in anthocyanin levels in mango (cv. Kent) with almost no effect on the compound composition (Figure 5). As a comparison, mango fruit exposed to sunlight showed an increase of the same flavonoids in comparison to fruit from the inside of the tree canopy [8]. Due to the increase in flavonols and anthocyanins, which have antioxidative properties, it has been observed that preharvest application with phenylalanine also improved antioxidant activity both in mango and apple fruit (Figure 3, Figure 4 and Figure 5). Indeed, a positive correlation between total phenolic and flavonoid content and the level of antioxidant activity was found in different apple varieties [39].

## 5. Conclusions

Preharvest treatment of Phenylalanine in combination with sunlight radiation increased the phenylpropanoid biosynthesis pathway, leading to an increase in the coverage and intensity of red-colored peel fruit in various mango and apple cultivars. Phe application increased the phenolic and flavonoid contents due to the activation of the phenylpropanoid pathway, which in combination with sunlight radiation led to the biosynthesis of anthocyanins that directly contribute to the red color of the peel and have health benefits. This study provides an effective new method of Phe spray at the orchard to improve the appearance of the fruit and its health benefits.

## 6. Patents

Provisional Patent Application No. 63/134,403 and 63/164,051, titled “Methods for improving fruit quality.”

## Data Availability

Data is contained within the article or supplementary material .

## Supplementary Materials

The following supporting information can be downloaded at: https://www.mdpi.com/article/10.3390/antiox11030491/s1.
