# Supplementary material for: Preharvest Application of Phenylalanine Induces Red Color in Mango and Apple Fruit’s Skin
Source: Antioxidants (Basel). 2022 Feb 28;11(3):491. doi: 10.3390/antiox11030491 (PMC8944447; doi:10.3390/antiox11030491)
Supplement: Supplementary file 1 [file antioxidants-11-00491-s001.zip › antioxidants-1589190-supplementary.pdf]

Preharvest application of phenylalanine induces red color in mango and apple fruit's skin

Michal Fanyuk[1,2], Manish Kumar Patel[1], Rinat Ovadia[3], Dalia Maurer[1], Oleg Feygenberg[1], Michal Oren-Shamir[3], Noam Alkan[1*]

1 Department of Postharvest Science of Fresh Produce, Agricultural Research Organization (ARO), Volcani Institute, Rishon LeZion 7505101, Israel; michal.fanyuk@mail.huji.ac.il (M.F); patelm1402@gmail.com (M.K.P); daliam@volcani.agri.gov.il (D.M); fgboleg@volcani.agri.gov.il (O.F).

2 Robert H. Smith Faculty of Agriculture, Food and Environment, The Hebrew University of Jerusalem, Rehovot, 76100, Israel; michal.fanyuk@mail.huji.ac.il (M.F).

3 Department of Plant Science, Agricultural Research Organization (ARO), Volcani Institute, Rishon LeZion 7505101, Israel; rinat@volcani.agri.gov.il (R.O); vhshamir@volcani.agri.gov.il (M.O.S).

* Correspondence: Noam Alkan, noamal@volcani.agri.gov.il; 972-3-9683605

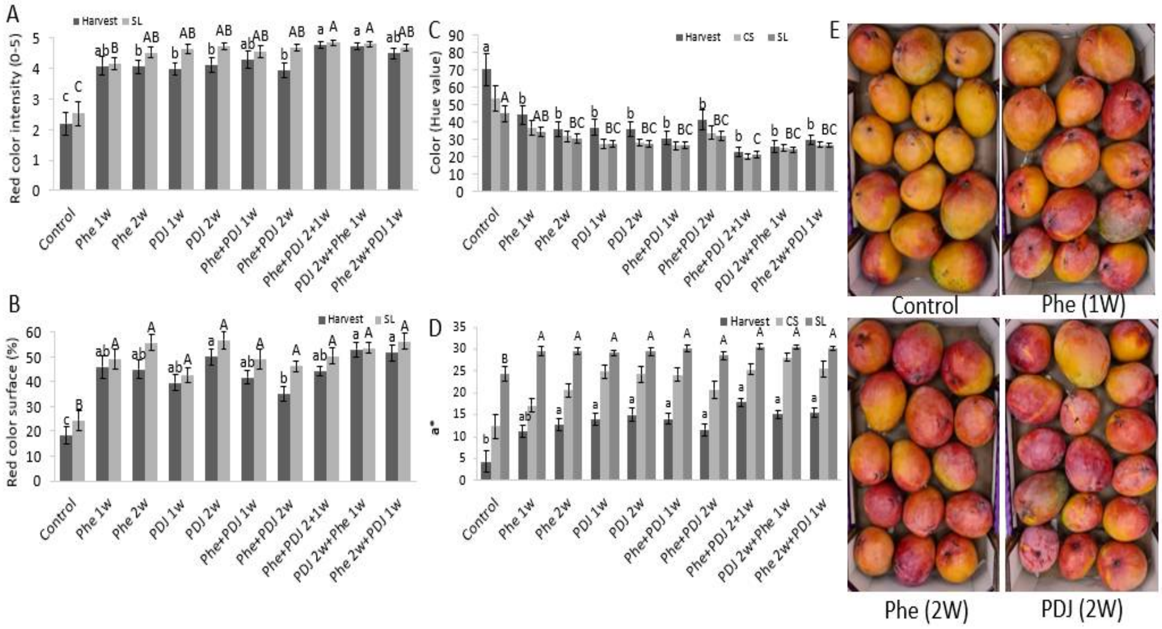

Figure S1. Red color evaluation of mango (cv. Shelly) peel. Shelly mango orchards were sprayed with 0.12% phenylalanine (Phe) or 0.2% prohydrojasmon (PDJ), one or two weeks preharvest. Different letters represent a significant difference (P≤ 0.05).

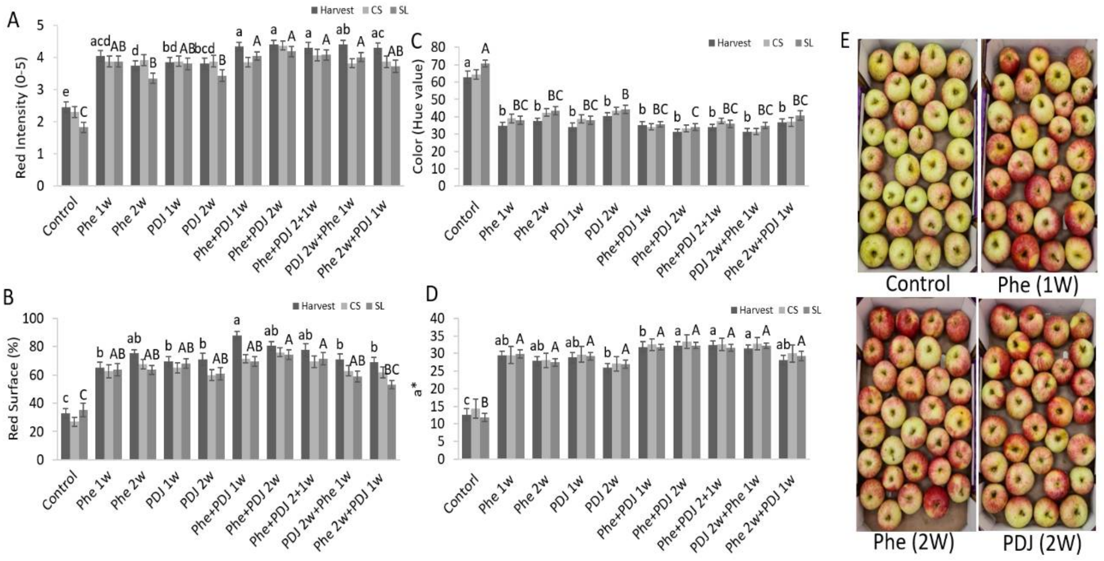

Figure S2. Red color evaluation of apple (cv. Gala) peel. The fruit was treated preharvest with spraying of 0.12% Phenylalanine and/or 0.2% prohydrojasmon (PDJ). The fruit was evaluated at 3-time points: at harvest (T0), after cold storage (2 months at 2°C), and shelf life (7 days at 22°C). Different letters represent a significant difference (P≤ 0.05).

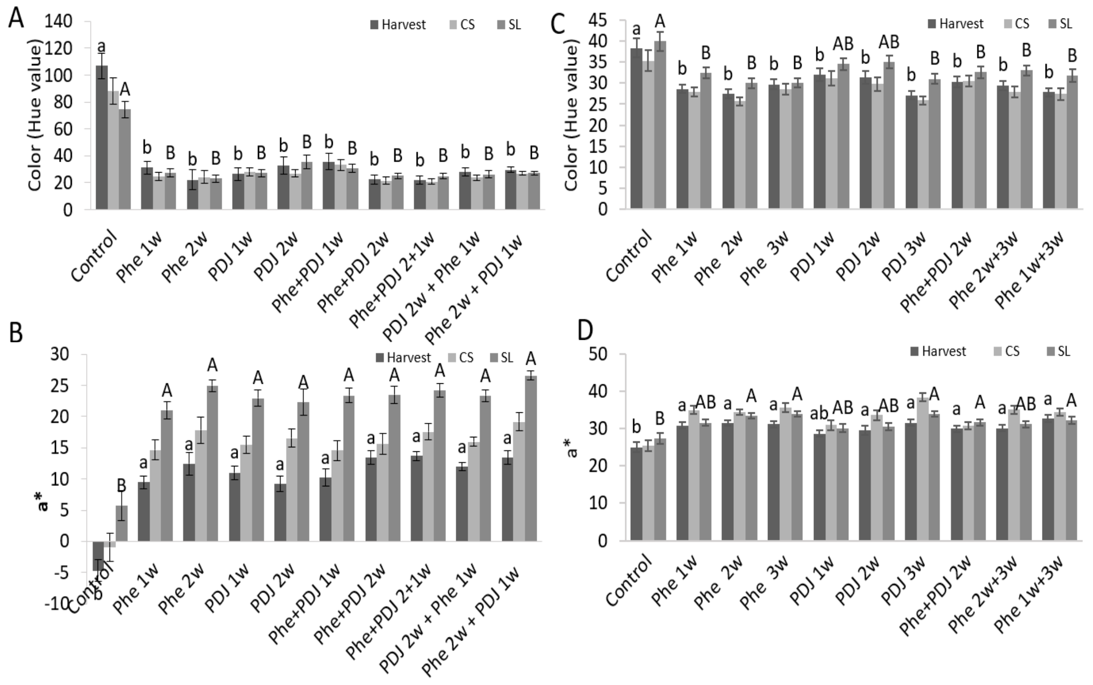

Figure S3. Quantification of red color parameters in ‘Kent’ mango and ‘Cripps pink’ apples. The fruit was treated preharvest with spraying of 0.12% Phenylalanine and/or 0.2% prohydrojasmon (PDJ). Different letters represent significant differences (P≤ 0.05).

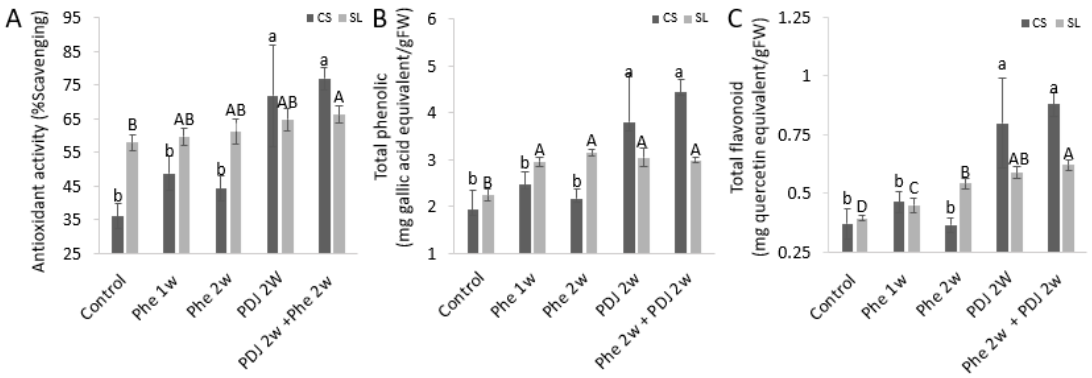

Figure S4. Effect of one or two weeks preharvest treatment on mango (cv. Shelly) fruit with 0.12% Phenylalanine or 0.2 % PDJ after harvest, cold storage (CS), and shelf-life (SL). A. Antioxidant activity, B. Total phenolic and C. Total flavonoid content was evaluated from mango peels (cv. Kent). Different letters represent significant differences (P≤ 0.05).

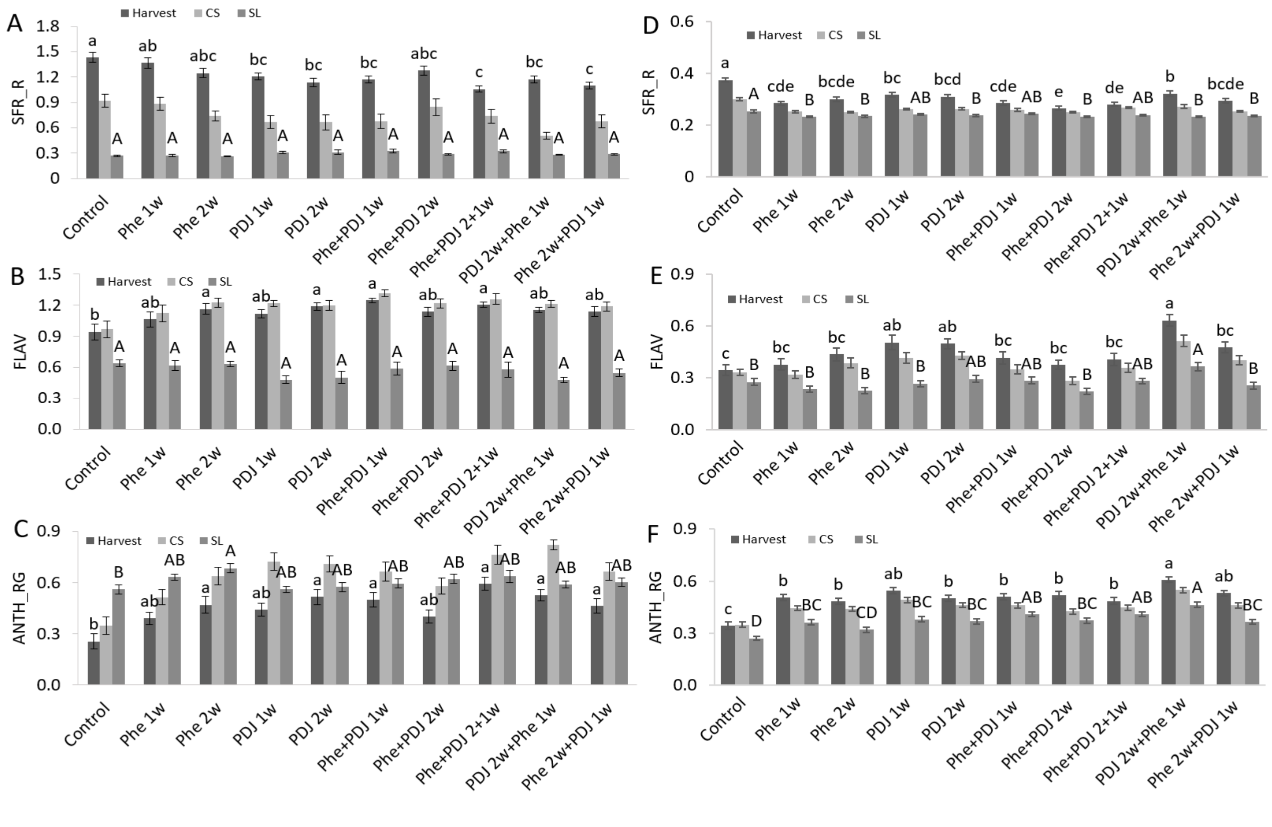

Figure S5. Fruit peel fluorescence. 'Shelly' mango and 'Galla' apple orchards were sprayed with 6mM phenylalanine (Phe) or 0.2% prohydrojasmon (PDJ), one or two weeks preharvest. Fruit peel fluorescence was measured at the reddest point of 'Shelly' mango (A-C) and 'Galla' apple (D-F) at harvest, after cold storage (CS), and after shelf life (SL). A, D. Chlorophyll (SFR_R). B, E. Flavonoids (FLAV). C, F. Anthocyanin (ANTH_RG). Different letters represent a significant difference (P≤0.05).

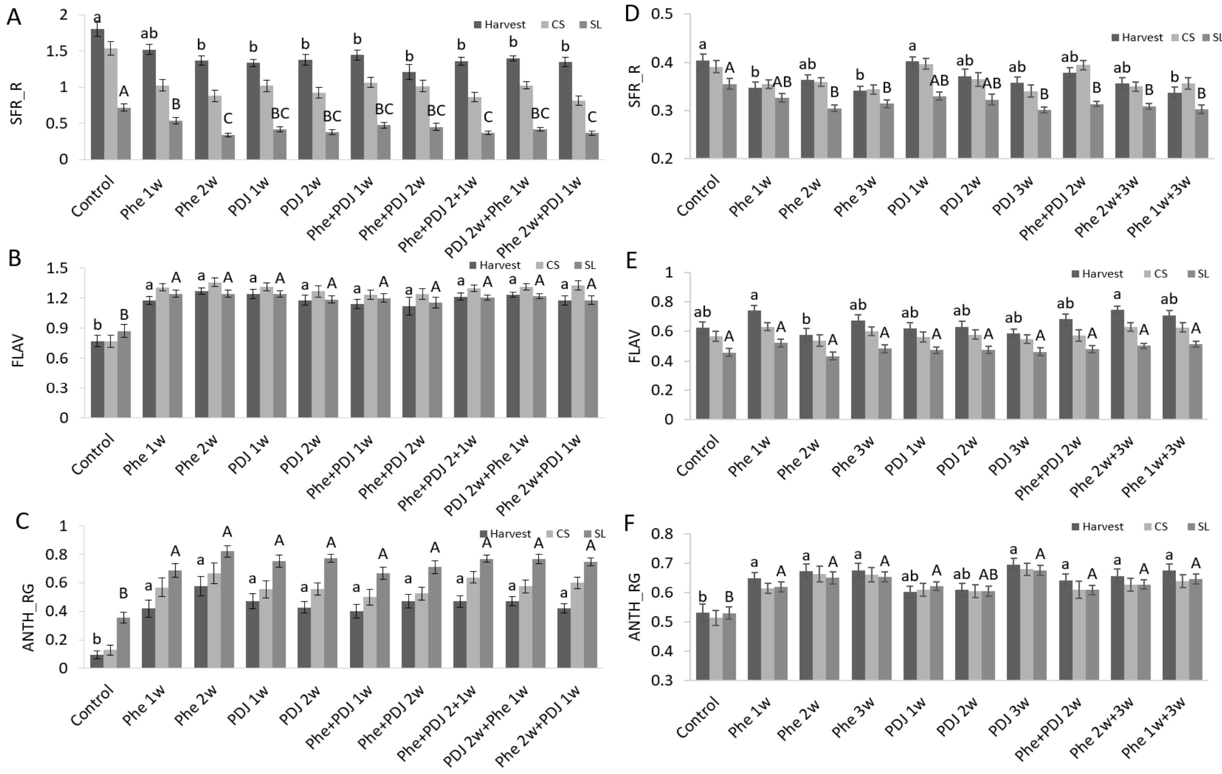

Figure S6. Fruit peel fluorescence. 'Kent' mango and 'Cripps pink' apple orchards were sprayed with 6mM phenylalanine (Phe) or 0.2% prohydrojasmon (PDJ), one or two weeks preharvest. Fruit peel fluorescence was measured at the reddest point of 'Kent' mango (A-C) and 'Cripps pink' apple (D-F) at harvest, after cold storage (CS), and after shelf life (SL). A, D. Chlorophyll (SFR_R). B, E. Flavonoids (FLAV). C, F. Anthocyanin (FER_RG). Different letters represent statistically significant differences (P≤0.05).

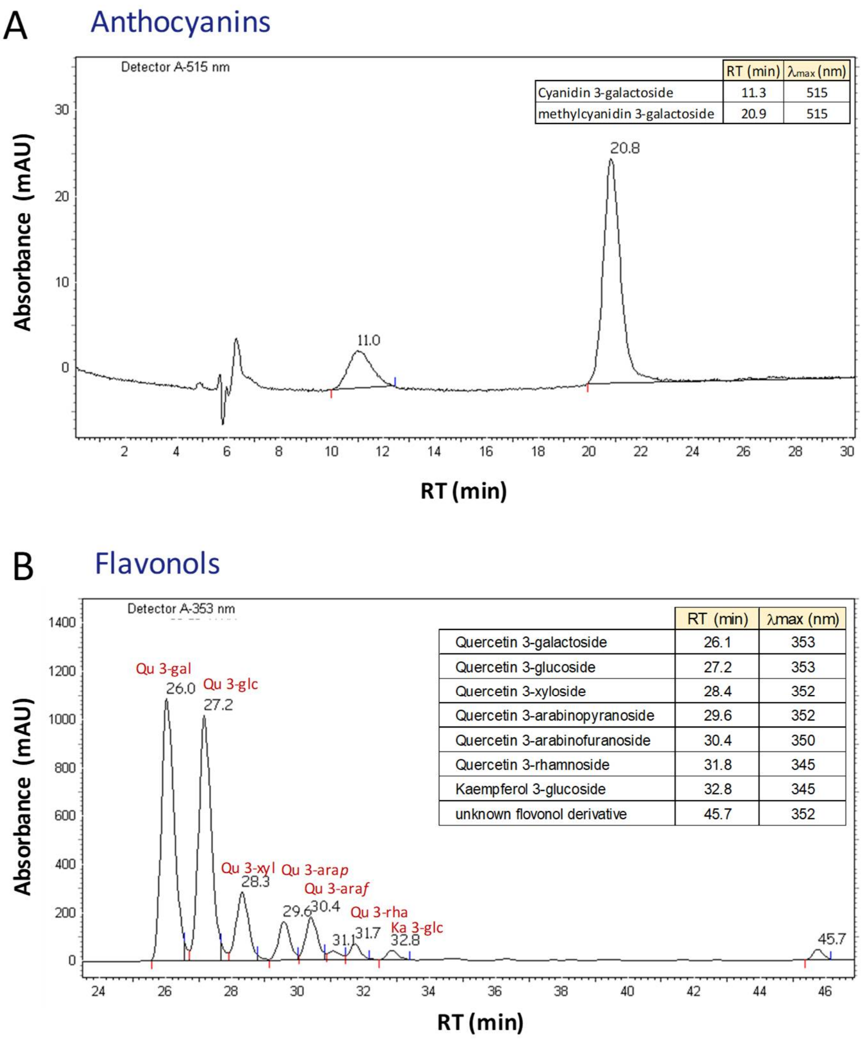

Figure S7. Chromatogram of mango (cv. Kent) fruit peel extract using HPLC. (A) Anthocyanins, (B) Flavonols.

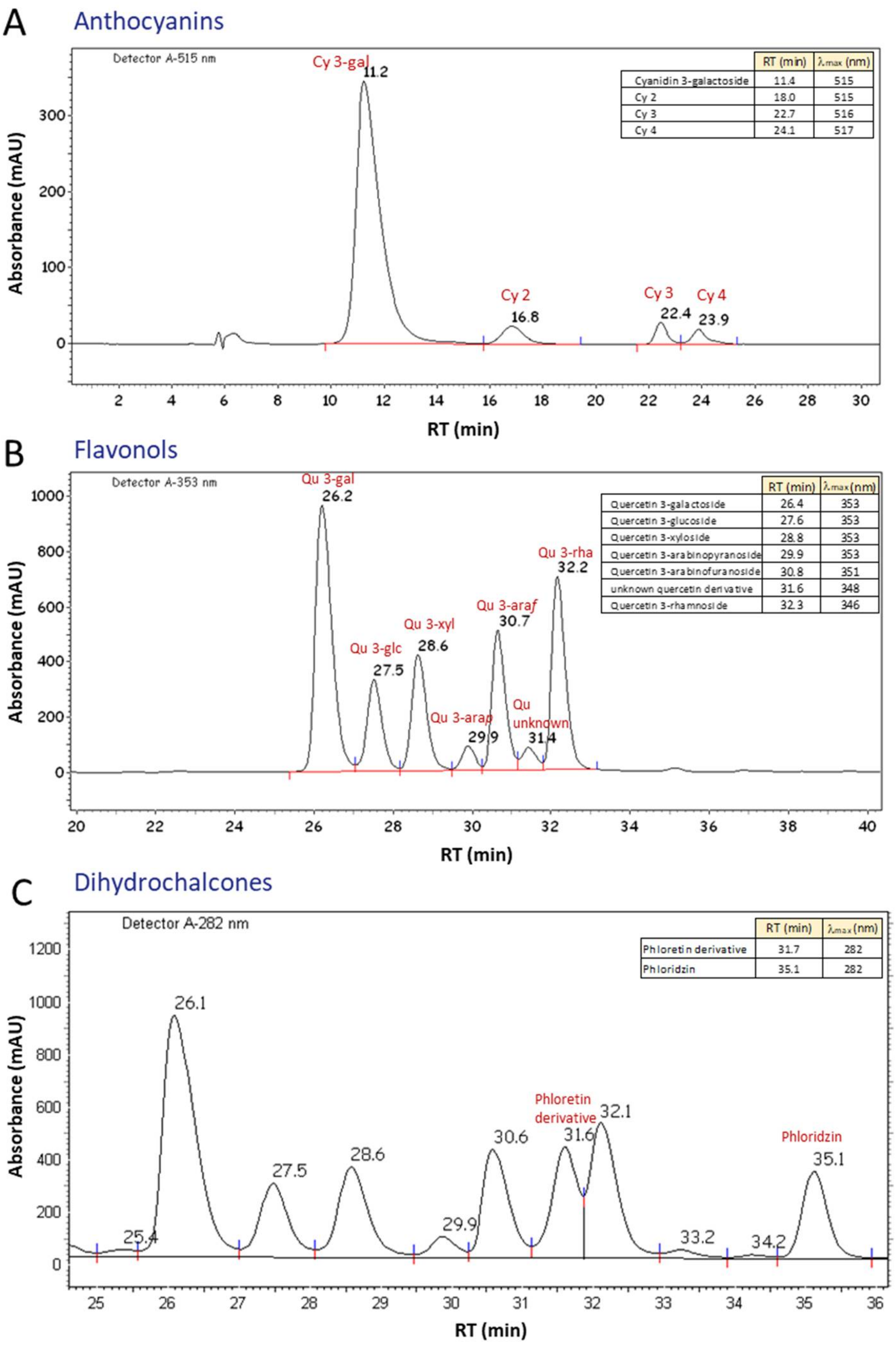

Figure S8. Chromatogram of apple (cv. Cripps pink) fruit peel extract using HPLC. (A) Anthocyanins, (B) Flavonols, (C) Dihydrochalcones.

Table S1. Effect of preharvest application of phenylalanine on the red color of mango (cv. 'Shelly') fruit (2021).

	Red surface (%)		Red intensity (index 0-5)		Color (hue)		a* value		SFR_R		FLAV		ANTH_RG	
	T0	SL	T0	SL	T0	SL	T0	SL	T0	SL	T0	SL	T0	SL
Control	34.39 ± 2.73c	32.27 ± 2.58b	2.53 ± 0.16d	2.45 ± 0.17d	67.29 ± 8.38a	32.81 ± 2.59a	5.54 ± 1.98b	33.45 ± 0.92a	1.13 ± 0.06a	0.36 ± 0.06a	0.85 ± 0.05b	0.54 ± 0.11a	0.33 ± 0.04c	0.49 ± 0.06a
Phe 2w 0.01%	35.89 ± 3.6c	36.41 ± 3.38b	2.31 ± 0.24d	2.81 ± 0.14d	70.50 ± 6.42a	31.85± 2.84ab	3.41 ± 1.04b	28.14 ± 2.23a	1.15 ± 0.07a	0.34 ± 0.05a	0.93 ± 0.07b	0.65 ± 0.12a	0.30 ± 0.04c	0.59 ± 0.07a
Phe 2w 0.06%	43.28 ± 3.48bc	58.75 ± 3.45a	3.50 ± 0.15c	4.33 ± 0.09b	17.02 ± 3.37b	23.47 ±2.81abc	18.88 ± 1.48a	29.60 ± 2.30a	0.95 ± 0.05abc	0.33 ± 0.03a	1.24 ± 0.06a	0.66 ± 0.13a	0.70 ± 0.06ab	0.58 ± 0.07a
Phe 2w 0.12%	55.57 ± 3.21ab	63.17± 2.51a	4.42 ± 0.12a	4.80 ± 0.09ac	22.63 ± 2.51b	22.21 ± 3.11abc	22.95 ± 2.69a	29.19 ±1.95a	0.88 ± 0.05bc	0.29 ± 0.01a	1.25 ± 0.04a	0.66 ± 0.07a	0.69 ± 0.03ab	0.68 ± 0.06a
Phe 2w 0.24%	58.13 ± 2.63a	60.37± 2.49a	4.34 ± 0.13ab	4.74 ± 0.11a	21.17 ± 3.62b	20.17 ± 3.22bc	23.18 ± 2.02a	31.70 ± 2.50a	0.75 ± 0.04c	0.27 ± 0.01a	1.26 ± 0.04a	0.53 ± 0.07a	0.75 ± 0.05a	0.61 ± 0.06a
Flowering + 2w 0.12%	57.30 ± 2.14a	63.76 ± 2.27a	4.11 ± 0.13bc	4.36 ± 0.08b	22.88 ± 4.23b	19.62 ±2.24c	16.45 ± 1.59a	28.48 ± 1.71a	1.06 ± 0.03bc	0.31 ± 0.03a	1.27 ± 0.03a	0.83 ± 0.09a	0.64 ± 0.03ab	0.75 ± 0.04a
Flowering (Phe 0.12%)	55.57± 2.05ab	60.68 ± 2.20a	4.11 ± 0.11abc	4.74 ± 0.10a	21.97 ± 3.43b	23.24 ± 2.97abc	17.53 ± 1.37a	32.11 ± 0.70a	1.01 ± 0.04ab	0.35 ± 0.04a	1.21 ± 0.05a	0.57± 0.1a	0.54 ± 0.05b	0.60 ± 0.07a
Phe 0.12% 2w+4w	57.29 ± 1.74a	59.38 ±1.90a	4.34 ± 0.17ab	4.59 ± 0.24abc	19.78 ± 3.22b	25.23 ± 1.80abc	19.30 ± 1.66a	31.61 ± 1.10a	0.88 ± 0.03ab	0.28 ± 0.02a	1.28 ± 0.04a	0.62 ± 0.13a	0.60 ± 0.03ab	0.59 ± 0.08a

Table S2. Effect of preharvest application of phenylalanine on the red color of mango (cv. 'Tommy Atkins') fruit.

	Red surface (%)			Red intensity (index 0-5)			Color (hue)		a* value		SFR_R		FLAV		ANTH_RG	
	T0	CS	SL	T0	CS	SL	T0	SL	T0	SL	T0	SL	T0	SL	T0	SL
Control	46.01 ± 1.94b	46.14 ± 2.5b	47.62 ± 2.18b	1.94 ± 0.10d	3.36 ± 0.19d	3.68 ± 0.12c	29.78 ± 12.27a	25.14 ± 7.03a	7.01 ± 2.32b	18.36 ± 2.26b	1.51 ± 0.08a	0.65 ± 0.09a	1.02 ± 0.05b	0.82 ± 0.07a	0.60 ± 0.09b	0.69 ± 0.05a
Phe 2w 0.06%	56.90 ± 2.77a	58.26 ± 4.11a	59.52 ± 3.65a	4.48 ± 0.12b	4.3 ± 0.15bc	4.23 ± 0.17b	11.98 ± 2.48a	16.55 ± 1.79a	9.66 ± 1.04ab	21.02 ± 1.05ab	1.41 ± 0.07ab	0.59 ± 0.08a	1.12 ± 0.06ab	0.84 ± 0.06a	0.70 ± 0.08ab	0.83 ± 0.03a
Phe 2w 0.12%	64.86± 1.65a	63.89 ± 2.93a	67.069 ± 1.80a	4.7 ± 0.08ab	4.72 ± 0.11a	4.79 ± 0.08a	4.20±3.14a	10.2 ± 1.54a	10.31 ± 0.71ab	21.89 ± 1.51ab	1.43 ± 0.08ab	0.46 ± 0.04a	1.13 ± 0.04ab	0.63 ± 0.06a	0.68 ± 0.06ab	0.78 ± 0.04a
Phe 2w 0.24%	60.52 ± 2.02a	65 ± 2.59a	66.43 ± 2.44a	4.62 ± 0.09b	4.61 ± 0.16ab	4.52 ± 0.13ab	10.11 ± 2.56a	21.43 ± 3.19a	8.66 ± 0.70ab	21.45 ± 2.29ab	1.28 ± 0.09ab	0.62 ± 0.09a	1.16 ± 0.05ab	0.91 ± 0.09a	0.70 ± 0.07ab	0.73 ± 0.06a
Flowering + 2w 0.12%	59.66 ± 2.34a	58.5 ± 2.74a	60.24 ± 2.2a	4.59 ± 0.12b	4.55 ± 0.14ac	4.62 ± 0.15ab	24.34 ± 4.70a	19.59 ± 3.1a	9.94 ± 1.32ab	21.86 ± 2.22ab	1.37 ± 0.06ab	0.55 ± 0.14a	1.17 ± 0.02ab	0.74 ± 0.08a	0.71 ± 0.05ab	0.70 ± 0.04a
Flowering (Phe 0.12%)	59.33 ± 1.45a	62.35 ± 2.19a	55 ± 2.26a	4.3 ± 0.09c	3.82 ± 0.13d	4.68 ± 0.10ab	11.38 ± 2.08a	18.03 ± 1.52a	13.72 ± 0.85a	27.78 ± 1.15a	1.28 ± 0.04ab	0.39 ± 0.02a	1.24 ± 0.03a	0.82 ± 0.07a	0.76 ± 0.05ab	0.74 ± 0.04a
Phe 0.12% 2w+4w	65.44 ± 1.48a	61.21 ± 2.07a	65 ± 2.1a	4.84 ± 0.05a	4.57 ± 0.1ac	4.68 ± 0.08a	5.57 ± 4.21a	25.34 ± 3.74a	10.93 ± 0.51ab	23.52 ± 1.38ab	1.15 ± 0.08b	0.45 ± 0.04a	1.16 ± 0.03ab	0.76 ± 0.07a	0.83 ± 0.07a	0.80 ± 0.04a

Table S3. Effect of preharvest application of phenylalanine on the red color of apple (cv. 'Cripps pink') fruit after 3w in CS (2021).

	Red surface (%)	Red intensity (index 0-5)	Color (hue)	a* value	SFR_R	FLAV	ANTH_RG
Control	50.53 ± 1.38f	2.35 ± 0.08f	39.64 ± 3.28a	25.39 ± 1.86d	0.34 ± 0.01a	0.35 ± 0.04b	0.44 ± 0.03b
Phe 0.01% 6W	56.18 ± 1.70ef	2.54 ± 0.10ef	35.67 ± 2.55abc	28.30 ± 1.55cd	0.34 ± 0.01a	0.50 ± 0.03ab	0.51 ± 0.03ab
Phe 0.01% 4W	58.66 ± 1.55cde	2.91 ± 0.09bc	32.12 ± 2.20abcd	30.96 ± 1.47abcd	0.30 ± 0.01ab	0.44± 0.03 ab	0.52 ± 0.02ab
Phe 0.01% 2W	58.38 ± 1.44de	2.47 ± 0.09ef	36.08 ± 2.27ab	27.80 ± 1.52cd	0.31 ± 0.01ab	0.38 ± 0.03ab	0.44 ± 0.03b
Phe 6w 0.12%	60.40 ± 1.32bcde	3.07 ± 0.09c	27.97 ± 1.75bcd	33.16 ± 1.19abc	0.32 ± 0.01ab	0.53 ± 0.03a	0.60 ± 0.03a
Phe 4w 0.12%	59.66 ± 1.28bcde	3.01 ± 0.10c	32.60 ± 2.96abcd	31.05 ± 1.89abcd	0.30 ± 0.01b	0.47 ± 0.04ab	0.53 ± 0.03ab
Phe 2w 0.12%	67.78 ± 1.29a	3.15 ± 0.08bc	24.31 ± 0.95d	37.00 ± 0.69a	0.28 ± 0.01b	0.50 ± 0.04ab	0.60 ± 0.03a
Phe 4+6w 0.12%	67.91 ± 1.09a	3.47 ± 0.07a	28.08 ± 1.50bcd	34.30 ± 1.11abc	0.29 ± 0.01b	0.47 ± 0.03ab	0.58 ± 0.03a
Phe 2+6w 0.12%	65.16 ± 1.40abc	3.40 ± 0.09a	25.38 ± 2.16cd	35.28 ± 1.37ab	0.29 ± 0.01b	0.53 ± 0.03a	0.62 ± 0.03a
Phe 2+4w 0.12%	63.59 ± 1.48abcd	3.38 ± 0.09ab	29.87 ± 2.14abcd	33.19 ± 1.48abc	0.30 ± 0.01b	0.44 ± 0.03ab	0.55 ± 0.02ab
Formulation 1 6W	65.85 ± 1.30ab	3.36 ± 0.08ab	27.31 ± 1.01bcd	36.28 ± 0.87ab	0.29 ± 0.01b	0.43 ± 0.03ab	0.57 ± 0.02ab
Formulation 1 4W	63.27 ± 1.44abcd	3.14 ± 0.09bc	34.17 ± 2.79abcd	30.08 ± 1.84bcd	0.30 ± 0.01ab	0.46 ± 0.04ab	0.53 ± 0.03ab
Formulation 1 2W	63.05 ± 1.06bcd	2.72 ± 0.09de	30.24 ± 1.32abcd	32.43 ± 1.17abc	0.31 ± 0.01ab	0.42 ± 0.03ab	0.53 ± 0.03ab

Table S4. Effect of preharvest application of phenylalanine on the red color of apple (cv. 'Starking Delicious) fruit.

	Red surface (%)	Red intensity (index 0-5)	Color (hue)	a* value	SFR_R	FLAV	ANTH_RG
Control	33.56 ± 17.16d	1.96 ± 1.01d	64.31 ± 3.60a	10.80 ± 1.50e	0.54 ± 0.01a	0.29 ± 0.05b	0.31 ± 0.02c
Phe 4w 0.01%	51.60 ± 22.16c	2.79 ± 1.15c	46.22 ± 3.08bc	18.56 ± 1.43cd	0.56 ± 0.01a	0.41 ± 0.05ab	0.37 ± 0.03c
Phe 2w 0.01%	56.30 ± 23.46c	3.06 ± 1.23c	49.39 ± 4.86b	17.69 ± 2.22cd	0.54 ± 0.01a	0.38 ± 0.05ab	0.39 ± 0.03c
Phe 2+4w 0.01%	42.16 ± 14.87d	2.76 ± 0.76c	51.85 ± 3.01b	16.26 ± 1.31de	0.54 ± 0.02a	0.45 ± 0.06ab	0.34 ± 0.02c
Phe 4w 0.12%	81.62 ± 14.87ab	4.60 ± 0.57ab	34.42 ± 1.72cd	24.97 ± 0.99ab	0.54 ± 0.01a	0.36 ± 0.05ab	0.54 ± 0.02b
Phe 2w 0.12%	77.85 ± 10.93b	4.40 ± 0.66b	35.25 ± 2.67b	22.61 ± 1.16ac	0.59 ± 0.01a	0.52 ± 0.05ab	0.53 ± 0.02b
Phe 2+4w 0.12%	81.74 ± 10.39ab	4.70 ± 0.57a	29.15 ± 1.23d	27.21 ± 0.83ab	0.53 ± 0.02a	0.46 ± 0.06ab	0.61 ± 0.02ab

Table S5. Effect of preharvest application of phenylalanine on the red color of apple (cv. 'Anna') fruit.

	Red surface (%)		Red intensity (index 0-5)		Color (hue)		a* value		SFR_R		FLAV		ANTH_RG	
	T0	SL	T0	SL	T0	SL	T0	SL	T0	SL	T0	SL	T0	SL
Control	24.09 ± 2.84b	31.54 ± 5.33b	1.70 ± 0.14b	2.54 ± 0.28b	68.38 ± 4.88a	62.52 ± 4.42a	7.03 ± 1.69b	12.84 ± 1.92b	0.53 ± 0.01a	0.52 ± 0.01a	0.81 ± 0.04a	0.65 ± 0.03a	0.30 ± 0.04b	0.37 ± 0.03b
Phe 4w 0.12%	56.55 ± 2.44a	70.75 ± 5.00a	3.33 ± 0.14a	4.05 ± 0.26a	36.06 ± 3.41b	35.73 ± 3.31b	20.10 ± 1.23a	25.01 ± 0.96a	0.41 ± 0.02b	0.38 ± 0.02c	0.76 ± 0.03a	0.60 ± 0.02a	0.59 ± 0.04a	0.54 ± 0.03a
Phe 2w 0.12%	49.06 ± 2.88a	54.78 ± 6.16a	3.27 ± 0.15a	3.70 ± 0.28a	46.93 ± 5.0b	41.85 ± 4.11b	15.80 ± 1.78a	20.99 ± 1.51a	0.47 ± 0.02ab	0.44 ± 0.01b	0.86 ± 0.04a	0.67 ± 0.03a	0.46 ± 0.04a	0.56 ± 0.03a
Phe 2+4w 0.12%	53.40 ± 2.64a	64.67 ± 4.47a	3.54 ± 0.15a	3.73 ± 0.25a	39.66 ± 4.10b	38.11 ± 2.81b	17.02 ± 1.45a	22.41 ± 1.14a	0.46 ± 0.02b	0.43 ± 0.02bc	0.74 ± 0.03a	0.58 ± 0.03a	0.52 ± 0.04a	0.54 ± 0.03a

Table S6. Effect of preharvest application of phenylalanine decay parameters in mango (cv. Kent, Shelly, and Tommy) fruit.

		SER		Side decay		Total rotten fruit
Cultivar	Treatment	Index	Decay (%)	Index	Decay (%)	Decay (%)
Mango (cv. Kent), 2020	Control	0.91 ± 0.50a	11.62 ± 3.28ab	0.11 ± 0.03a	11.36 ± 2.66ab	22.98 ± 3.22ab
	1W Phe	0.05 ± 0.03a	5.41 ± 2.76b	1.30 ± 0.72a	20.52 ± 3.76a	20.52 ± 3.76ab
	2W Phe	0.43 ± 0.24a	5.81 ± 2.91b	0.36 ± 0.28a	8.37 ± 0.40ab	14.18 ± 3.27b
	1W PDJ	0.45 ± 0.09a	10.83 ± 2.10	0.21 ± 0.21a	2.08 ± 2.08b	12.92 ± 3.49b
	2W PDJ	1.37 ± 0.23a	16.74 ± 0.81ab	1.51 ± 0.21a	19.31 ± 1.93a	36.05 ± 1.49a
	1W Phe+PDJ	0.48 ± 0.24a	4.76 ± 2.38ab	0.83 ± 0.17a	21.43 ± 4.12a	23.81 ± 6.3ab
	2W Phe+PDJ	0.42 ± 0.24a	5.56 ± 2.78b	0.28 ± 0.28a	2.78 ± 2.78b	8.33 ± 4.81b
	2+1W Phe+PDJ	0.28 ± 0.25a	4.95 ± 2.48b	0.58 ± 0.46a	14.65 ± 4.14ab	19.60 ± 2.70ab
	2W PDJ +1W Phe	1.82 ± 0.18a	19.41 ± 2.01ba	0.02 ± 0.02a	2.38 ± 2.38b	21.79 ± 3.81ab
	2W Phe +1W PDJ	0.63 ± 0.15a	9.73 ± 2.83ab	0.42 ± 0.19a	12.11 ± 2.74ab	19.46 ± 5.66ab
Mango (cv. Shelly), 2020	Control	0.00 ± 0.00a	5.56 ± 0.00b	0.06 ± 0.03a	0.00 ± 2.94a	5.56 ± 2.94a
	1W Phe	0.02 ± 0.02a	0.00 ± 2.08ab	0.00 ± 0.00a	2.08 ± 0.00a	2.08 ± 2.08a
	2W Phe	1.16 ± 0.29a	2.56 ± 3.14a	0.26 ± 0.26a	12.91 ± 2.56a	15.47 ± 4.77a
	1W PDJ	1.18 ± 0.26a	0.00 ± 2.63ab	0.00 ± 0.00 a	11.79 ± 0.00a	11.79 ± 2.63a
	2W PDJ	0.99 ± 0.15a	0.00 ± 1.55ab	0.00 ± 0.00a	9.88 ± 0.00a	9.88 ± 1.55a
	1W Phe+PDJ	0.15 ± 0.15a	0.00 ± 3.03ab	0.00 ± 0.00a	3.03 ± 0.00a	3.03 ± 3.03a
	2W Phe+PDJ	0.30 ± 0.27a	4.95 ± 2.68ab	0.14 ± 0.11a	5.34 ± 2.48a	10.29 ± 2.57a
	2+1W Phe+PDJ	0.65 ± 0.34a	4.46 ± 2.76a	0.33 ± 0.18a	12.10 ± 2.25a	14.19 ± 3.57a
	2W PDJ +1W Phe	0.82 ± 0.13a	2.56 ± 1.97ab	0.26 ± 0.26a	11.49 ± 2.56a	14.06 ± 0.84a
	2W Phe +1W PDJ	0.22 ± 0.22a	8.35 ± 2.22ab	0.08 ± 0.02a	2.22 ± 2.09a	10.57 ± 2.36a
Mango (cv. Shelly), 2021	Control	-	-	4.78 ± 0.92ab	92.71 ± 4.29a	-
	Phe 0.01% 2w	-	-	4.30 ± 0.52ab	78.13 ± 8.57ab	-
	Phe 0.06% 2w	-	-	4.06 ± 0.89ab	88.89 ± 5.56ab	-
	Phe 0.12% 2w	-	-	3.67 ± 1.02ab	85.00 ± 7.64ab	-
	Phe 0.24% 2w	-	-	4.02 ± 0.74a	82.50 ± 6.85ab	-
	Phe 0.12% 2+4w	-	-	1.62 ± 0.25b	60.32 ± 3.17b	-
	Phe 0.12% 2w + flower	-	-	4.54 ± 0.23a	92.26 ± 4.49a	-
	Phe 0.12% flower	-	-	4.92 ± 0.92ab	81.55 ± 6.76ab	-
Mango (cv. Tommy), 2021	Control	0.18 ± 0.13a	7.16 ± 3.00a	0.40 ± 0.07ab	34.73 ± 0.13a	40.76 ± 5.54a
	Phe 0.06% 2W	0.00 ± 0.00a	0.00 ± 0.00a	0.39 ± 0.07b	28.81 ± 0.00a	28.81 ± 3.14a
	Phe 0.12% 2W	0.03 ± 0.03a	3.13 ± 3.13a	0.03 ± 0.03c	3.13 ± 0.03a	6.25 ± 3.61a
	Phe 0.24% 2W	0.05 ± 0.04a	4.76 ± 4.12a	0.15 ± 0.01d	15.08 ± 0.04a	15.08 ± 0.79a
	Phe 0.12% 2+4W	0.05 ± 0.05a	5.00 ± 5.00a	0.16 ± 0.03cd	16.18 ± 0.05a	18.68 ± 4.60a
	Phe 0.12% Flower+ 2W	0.00 ± 0.00a	0.00 ± 0.00a	0.14 ± 0.08abcd	14.29 ± 0.00a	14.29 ± 8.25a
	Phe 0.12% Flower	0.00 ± 0.00a	0.00 ± 0.00a	0.18 ± 0.04ad	17.86 ± 0.00a	17.86 ± 3.57a

Table S7. Effect of preharvest application of phenylalanine decay parameters in apple (cv. Gala, Cripps pink and Starking Delicious) fruit.

		CS		SL	
Cultivar	Treatment	Index	Decay (%)	Index	Decay (%)
Apple (cv. Gala), 2020	Control	0.40 ± 0.13a	14.85 ± 3.72a	1.33 ± 0.34a	26.52 ± 2.05a
	1W Phe	0.37 ± 0.06a	8.89 ± 1.92a	1.15 ± 0.04a	21.34 ± 1.62a
	2W Phe	0.89 ± 0.15a	19.28 ± 3.60a	1.62 ± 0.42a	23.79 ± 3.10a
	1W PDJ	0.72 ± 0.09a	16.47 ± 3.09a	1.48 ± 0.16a	26.90 ± 2.73a
	2W PDJ	0.80 ± 0.30a	14.35 ± 3.88a	1.83 ± 0.35a	24.71 ± 3.84a
	1W Phe+PDJ	1.15 ± 0.33a	23.67 ± 5.61a	2.09 ± 0.52a	28.54 ± 9.00a
	2W Phe+PDJ	0.61 ± 0.12a	19.08 ± 3.44a	1.24 ± 0.28a	19.18 ± 3.24a
	2+1W Phe+PDJ	1.17 ± 0.14a	25.50 ± 7.05a	2.06 ± 0.10a	28.66 ± 1.92a
	2W PDJ +1W Phe	0.65 ± 0.10a	14.35 ± 1.25a	1.24 ± 0.11a	23.17 ± 3.56a
	2W Phe +1W PDJ	1.03 ± 0.21a	24.55 ± 4.91a	2.16 ± 0.44a	34.68 ± 4.93a
Apple (cv. Cripps pink), 2020	Control	-	-	0.39 ± 0.04a	9.49 ± 1.71ab
	Phe 1W	-	-	0.69 ± 0.26a	13.06 ± 1.68ab
	Phe 2W	-	-	0.02 ± 0.01a	2.08 ± 1.47c
	Phe 3W	-	-	0.12 ± 0.09a	3.33 ± 1.67bc
	PDJ 1W	-	-	0.22 ± 0.01a	5.51 ± 1.23abc
	PDJ 2W	-	-	0.17 ± 0.05a	5.02 ± 0.43bc
	PDJ 3W	-	-	0.15 ± 0.05a	5.18 ± 1.42bc
	Phe+PDJ 2W	-	-	0.36 ± 0.06a	6.53 ± 1.18abc
	Phe 2W+3W	-	-	0.27 ± 0.10a	6.79 ± 0.93abc
	Phe 1W+3W	-	-	0.44 ± 0.08a	8.67 ± 0.86abc
Apple (cv. Starking Delicious), 2021	Control	-	-	0.71 ± 0.20a	15.42 ± 4.7a
	Phe 2w 0.12%	-	-	0.52 ± 0.06a	8.36 ± 0.9a
	Phe 4w 0.12%	-	-	0.77 ± 0.23a	16.52 ± 2.23a
	Phe 2+4w 0.12%	-	-	0.86 ± 0.02a	10.87 ± 0.67a
	Phe 2w 0.01%	-	-	1.72 ± 1.25a	23.70 ± 15.19a
	Phe 4w 0.01%	-	-	1.57 ± 1.36a	19.85 ± 14.30a
	Phe 2+4w 0.01%	-	-	1.20 ± 0.36a	13.60 ± 5.15a
	Formulation 2 2+4W	-	-	0.70 ± 0.45a	10.48 ± 2.64a
	Formulation 1 2+4W	-	-	1.44 ± 0.06a	17.95 ± 1.28a
	Formulation 3 2+4W	-	-	0.58 ± 0.48a	8.19 ± 5.02a
